# Association of early cardiac rehabilitation on mortality in patients with dilated cardiomyopathy using national inpatient database

**DOI:** 10.1038/s41598-025-20593-7

**Published:** 2025-10-24

**Authors:** Yuichi Yasufuku, Yuichi Nishioka, Hideo Yasunaga, Tomoaki Imamura

**Affiliations:** 1 Department of Biostatistics and Data Science, Graduate School of Medicine, The University of Osaka, Suita, Japan; 2 Department of Public Health, Health Management and Policy, Nara Medical University, Kashihara, Japan; 3 Department of Clinical Epidemiology and Health Economics, School of Public Health, The University of Tokyo, Tokyo, Japan; 4 Department of Biostatistics and Science, Graduate School of Medicine, The University of Osaka, Center of Medical Innovation and Translational Research, 2-2 Yamadaoka, Suita, 565-0871 Osaka Japan

**Keywords:** Dilated cardiomyopathy, Acute heart failure, Cardiac rehabilitation, Propensity score, Epidemiology, Heart failure, Rehabilitation

## Abstract

Dilated cardiomyopathy (DCM) is a complex disease characterized by cardiomyopathic hypokinesis and left ventricular interior hypertrophy. Cardiac rehabilitation (CR) is an established treatment for some cardiovascular diseases; however, the outcomes of early CR for patients with DCM remain underexplored. This study aimed to investigate the association between early CR and 90-day mortality in patients with DCM and symptomatic heart failure, using a national inpatient database in Japan from July 1, 2010, to March 31, 2020. We applied multiple imputation to the missing data and propensity score matching analysis. Early CR was defined as that initiated within 3 days of admission. The study included 30,296 eligible patients, including those with early CR (*n* = 3,130) and delayed or no CR (*n* = 27,166). The early CR group showed significantly lower 90-day mortality compared to the delayed or no CR group (odds ratio, 0.70, 95% confidence interval, 0.53–0.93; *P* = 0.01). Compared to the delayed or no CR group, the early CR group exhibited a higher activities of daily living score at discharge, but there was no significant difference in length of stay between the groups.

## Introduction

Dilated cardiomyopathy (DCM) is a complex disease characterized by cardiomyopathic hypokinesis and left ventricular interior hypertrophy; some cases progress to chronic heart failure (HF) and recurrent acute exacerbations^1,2^. Reports from Western nations estimate the prevalence of DCM in adults to range between 0.036% and 0.40%^3^, whereas the annual incidence of chronic idiopathic DCM is reported to be 5–8 cases per 100,000 individuals, with concerns about potential underestimation^4^. In Japan, a study in 1998 estimated approximately 17,700 patients with DCM, corresponding to a prevalence of 14.0 per 100,000 individuals. Cohort studies from Japan suggested that patients with HF due to DCM constitute approximately 15–27% of HF cases^5–11^.

Cardiac rehabilitation (CR) is an established treatment for some cardiovascular diseases and comprises supervised physical activity, nutritional education, weight management, and lifestyle modifications^12–14^. CR aims to improve functional capacity, exercise duration, and Health-Related Quality of Life (HRQoL), decrease symptoms, mitigate cardiovascular risk factors, and enhance longevity and well-being by preventing the onset and exacerbation of cardiovascular diseases and other life-threatening events. CR improves physical performance and lowers patient mortality, especially in patients with myocardial infarction and chronic HF. In-hospital CR refers to a comprehensive program prescribed early after hospitalization for patients with acute cardiovascular disease to improve prognosis, including early independence in the Activities of Daily Living (ADL) and prevention of re-hospitalization. The evidence of in-hospital CR for acute coronary syndrome and chronic HF is largely established; evidence for in-hospital CR including early mobilization in the acute setting has also accumulated in recent years. The standard in-hospital CR program in Japan for patients with acute HF in the acute phase after hospitalization is to first introduce an early mobilization program under a risk management protocol to prevent disuse syndrome and promote independence in the hospital ward. If the patient is able to walk more than 300 m in a 6-minute walk test, the CR program is shifted to a comprehensive CR program centered on exercise therapy. Thereafter, based on the results of a re-evaluation of the patient’s general condition, the frequency, intensity, and time of endurance training are gradually increased, strength training is introduced, and education on lifestyle and prevention of exacerbation of HF is provided^15^. CR for patients with DCM and HF symptoms is still controversial, and the appropriateness of early intervention post-hospitalization remains a subject of debate. The 2023 European Society of Cardiology guidelines on cardiomyopathies conditionally recommend introducing moderate- to high-intensity exercise in patients with DCM to improve functional capacity, ventricular function, and HRQoL. However, these guidelines advise against high-intensity exercise in patients with symptomatic left ventricular systolic dysfunction or exercise-induced arrhythmias^3^. Similarly, the 2021 European Society of Cardiology guidelines on HF treatment recommend exercise therapy for all patients with HF to improve exercise capacity and HRQoL and reduce hospitalizations for HF, with supervised CR programs particularly emphasized for more severe, frail patients or patients with comorbidity. The latest Japanese HF guidelines (2018 revision) do not provide DCM-specific recommendations for exercise therapy but endorse programs targeting chronic HF, particularly for patients with HF with reduced ejection fraction^16^. However, most of the evidence supporting these recommendations is based on studies involving patients with chronic HF; only case reports and a few observational studies have reported the effectiveness of CR in patients with DCM presenting with acute HF symptoms. In Japan, early- and delayed-design studies using real-world data to investigate the effectiveness of CR in patients with cardiovascular diseases have increased. These studies targeted various populations, including patients who underwent post-cardiac surgery^17^ and patients with acute HF, with or without admission to intensive care units^18–20^. However, research on CR specifically for DCM is limited; the evidence comprises mainly case reports and small-sample studies^21–24^. Advancements in the understanding and treatment of DCM, as well as in early rehabilitation for critically ill patients, have been documented. However, the feasibility of introducing even earlier rehabilitation for patients with DCM and acute HF symptoms remains underexplored. This study aimed to investigate the association of early CR with 90-day mortality in patients with DCM and symptomatic HF, using a national inpatient database in Japan.

## Methods

### Data source

This retrospective cohort study used the Diagnosis Procedure Combination (DPC) database in Japan, which includes administrative claims and discharge information^25,26^. Participation in this database is mandatory for all 82 academic hospitals. It also receives voluntary contributions from more than 1,700 middle-to-large hospitals. This database covers approximately half of all acute care inpatients in Japan. It includes information on unique identifiers (encrypted for privacy), patient age, sex, body weight, height, admission and discharge dates, diagnoses (coded according to the International Classification of Diseases, 10th revision (ICD-10)), surgical and nonsurgical procedures (coded with Japanese original codes), prescribed medications, and discharge status. A previous study reported that the sensitivity (78.9%) and specificity (93.2%) were both high for recorded diagnoses^27^. The database distinguishes between comorbidities present at admission and post-admission complications. In addition, we used the 2015 Hospital Bed Function Report to include the degree of medical function fulfillment of each medical institution where the subject patients were hospitalized in this study^28^. The Hospital Bed Function Report is an all-inclusive dataset that records hundreds of pieces of information, such as hospital bed volume and the number of patients, which the Ministry of Health, Labour and Welfare began collecting from medical institutions nationwide in 2014. In general, the larger the hospital bed volume, the more advanced each function tends to be as a medical institution. This study received approval from The University of Tokyo’s Institutional Review Board [name of the ethics committee: The University of Tokyo, Clinical Research Review Board, approval number: 3501-(5) (May 7, 2021)]. As the data are anonymized, the requirement for informed consent was waived by the University of Tokyo’s Institutional Review Board. All aspects of the study adhered to the relevant guidelines and regulations. This study compiled DPC data from July 1, 2010, to March 31, 2020, and combined them with the 2015 Hospital Bed Function Report.

### Population

The study population comprised patients diagnosed with DCM, as recorded in the DPC database. The inclusion criteria were a diagnosis of DCM (ICD-10 code: I42.0) and HF (I110, I130, I132, I500, I501, or I509) classified as classifications Ⅱ–Ⅳ according to the New York Heart Association (NYHA) classification. We excluded patients aged under 18 years and those who died within 3 days of admission. The patients were divided into early CR and delayed or no CR groups based on whether CR was initiated within 3 days of admission. To eliminate immortal time bias, we excluded patients who died within 3 days of admission.

### Outcomes and covariates

The study’s primary outcome was 90-day mortality after admission. Secondary outcomes included the ADL score at discharge and length of stay. Covariates (baseline characteristics) included sex, age, NYHA classification, impaired consciousness at admission, body mass index (kg/m^2^), Charlson comorbidity index^29^, smoking index, and the ADL score at admission. The covariates also included ambulance use, cardiorespiratory assist devices, lifesaving procedures, primary admission to the intensive care unit or coronary care unit within 3 days of admission, hospital bed volume, and hospital codes.

### Statistical analysis

Missing data were handled using the multiple imputation method^30,31^. Continuous, binary, and ordinal outcomes with missing data were estimated using predictive mean matching, logistic regression, and polynomial regression. After multiple imputation, we used a one-to-one propensity score matching to account for differences in the observed factors that might affect the treatment assignment or outcome. The propensity score was defined as the probability of a patient undergoing early CR based on baseline covariates. Covariate selection was pre-specified using potential confounding factors and proxy variables for unknown or unmeasured confounding variables. The propensity score was estimated using a binary logistic regression model; the dependent variable was receiving early CR, and the independent variables were sex, age, NYHA classification, consciousness, body mass index, Charlson comorbidity index^7–11,32^, smoking index, ADL score at admission, ambulance use, cardiorespiratory assist devices, lifesaving procedures, primary admission to the intensive care unit or coronary care unit within 3 days after admission, hospital volume, and hospital codes. After estimating the propensity score, comparators were selected using the nearest-neighbor matching method in each of the 20 datasets^33–37^. Covariate balancing between the two groups in each imputed and matched dataset was assessed using the absolute standard mean difference with a cutoff set at < 0.1. For each of the 20 imputed and matched datasets, we estimated the odds ratios (OR) for binary outcomes (90-day mortality) and mean difference for continuous outcomes (ADL score at discharge and length of stay). The within-group method was used as the primary analysis for propensity integration, estimating the average treatment effect on the treated for each imputation and combining them using Rubin’s rule to produce a pooled mean difference or pooled OR^38^.

Stratified analyses were conducted using NYHA classifications Ⅱ, Ⅲ, and Ⅳ categories to assess the heterogeneity of effects across NYHA classification subgroups. Additionally, the definition of early CR was changed to within 2 days of admission to check for variability in results due to different timing of early rehabilitation initiation.

All statistical analyses were performed using R version 3.6.0 software. The mice and MatchThem packages were used for myocardial infarction and subsequent propensity score analysis. A two-tailed significance of *P* < 0.05 and 95% confidence intervals (CIs) were used in the analyses.

## Results

**Fig. 1:**
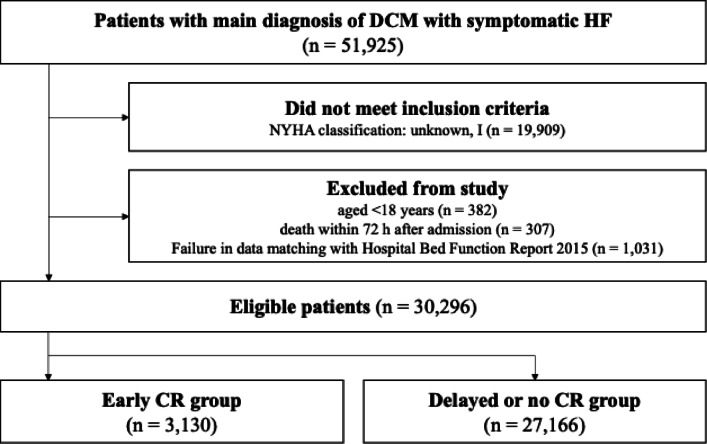
Flow chart of patients. We screened all patients admitted with DCM (ICD-10 code: I42.0) and symptomatic HF (I11.0, I13.0, I13.2, I50.0, I50.1, or I50.9). After excluding patients classified as NYHA classification Ⅰ or unknown, we further excluded those aged < 18 years, those who died within 72 h after admission, and cases where data could not be matched with the 2015 Hospital Bed Function Report. Finally, 30,296 patients with DCM and symptomatic HF were identified (early CR group, *n* = 3,130; delayed or no CR group, *n* = 27,166).

The study included 30,296 patients in the early CR group (*n* = 3,130) and the delayed or no CR group (*n* = 27,166). The patient selection flowchart is presented in Fig. 1. Table 1 shows the patients’ baseline characteristics before multiple imputation and propensity score matching. Notably, 27% of the patients in the delayed or no CR group received CR (Table 2). After multiple imputation and propensity score matching, all covariates had an absolute standard mean difference of < 0.1 within the groups for all 20 imputed datasets (Table 3). The 90-day mortality rate was significantly lower in the early CR group compared to the delayed or no CR group (OR: 0.70, 95% CI: 0.53–0.93; *P* = 0.01). Compared to the delayed or no CR group, the early CR group exhibited a greater ADL score at discharge (difference: 0.43, 95% CI: 0.08–0.78; *P* = 0.02), but no significant difference was found for length of stay (difference: −2.1 days, 95% CI: −4.7 to 0.5 days; *P* = 0.11) (Tables 4 and 5).

**Table 1:** Patient characteristics before propensity score matching and multiple imputation.

	Overall *n* = 30,296	Delayed or no CR *n* = 27,166	Early CR *n* = 3,130	*P*-value
Sex, n (%)				< 0.001
Men	21,516 (71%)	19,378 (71%)	2,138 (68%)	
Women	8,780 (29%)	7,788 (29%)	992 (32%)	
Age, year, mean (SD)	66.7 (14.3)	66.5 (14.3)	68.5 (14.0)	< 0.001
Age, year, n (%)				< 0.001
18–64	11,875 (39%)	10,796 (40%)	1,079 (34%)	
65–69	3,937 (13%)	3,519 (13%)	418 (13%)	
70–74	4,268 (14%)	3,856 (14%)	412 (13%)	
75–79	4,284 (14%)	3,801 (14%)	483 (15%)	
80–84	3,435 (11%)	3,039 (11%)	396 (13%)	
85–89	1,882 (6.2%)	1,612 (5.9%)	270 (8.6%)	
≥ 90	615 (2.0%)	543 (2.0%)	72 (2.3%)	
NYHA classification, n (%)				0.5
Ⅱ	8,107 (27%)	7,258 (27%)	849 (27%)	
Ⅲ	12,494 (41%)	11,184 (41%)	1,310 (42%)	
Ⅳ	9,695 (32%)	8,724 (32%)	971 (31%)	
Impaired consciousness, n (%)	28,127 (93%)	25,242 (93%)	2,885 (92%)	0.14
Body mass index, kg/m^2^, n (%)				0.011
< 18.5	4,277 (15%)	3,828 (15%)	449 (15%)	
18.5–24.9	16,182 (55%)	14,420 (55%)	1,762 (57%)	
≥ 25.0	8,993 (31%)	8,123 (31%)	870 (28%)	
Missing, n	844	795	49	
Charlson comorbidity index, n (%)				0.3
2	22,355 (74%)	20,063 (74%)	2,292 (73%)	
3	5,459 (18%)	4,866 (18%)	593 (19%)	
≥ 4	2,482 (8.2%)	2,237 (8.2%)	245 (7.8%)	
Smoking index, mean (SD)	319 (761)	320 (785)	308 (502)	0.415
Missing, n	3	3	0	
ADL score, mean (SD)	14.86 (7.25)	14.88 (7.25)	14.69 (7.27)	0.19
Ambulation, n (%)	7,454 (25%)	6,629 (24%)	825 (26%)	0.037
Missing, n	4,630	4,110	5,20	
Unexpected admission, n (%)	18,086 (60%)	15,840 (58%)	2,246 (72%)	< 0.001
Pharmaceuticals within 3 days of admission, n (%)				
Carperitide	10,312 (34%)	9,259 (34%)	1,053 (34%)	0.6
Nitrate	3,871 (13%)	3,423 (13%)	448 (14%)	0.007
Nicorandil	330 (1.1%)	306 (1.1%)	24 (0.8%)	0.081
Ca-channel blocker	1,066 (3.5%)	963 (3.5%)	103 (3.3%)	0.5
Dopamine	2,796 (9.2%)	2,623 (9.7%)	173 (5.5%)	< 0.001
Dobutamine	6,926 (23%)	6,205 (23%)	721 (23%)	0.8
Milrinone	1,031 (3.4%)	928 (3.4%)	103 (3.3%)	0.8
Noradrenaline	620 (2.0%)	576 (2.1%)	44 (1.4%)	0.009
Adrenaline	145 (0.5%)	134 (0.5%)	11 (0.4%)	0.3
Pimobendane	3,903 (13%)	3,454 (13%)	449 (14%)	0.011
Furosemide	16,562 (55%)	14,881 (55%)	1,681 (54%)	0.3
Tolvaptan	4,345 (14%)	3,636 (13%)	709 (23%)	< 0.001
Procedures within 3 days of admission, n (%)				
Intra-aortic balloon pumping	162 (0.5%)	155 (0.6%)	7 (0.2%)	0.017
Endotracheal mechanical ventilation	3,720 (12%)	3,193 (12%)	527 (17%)	< 0.001
Oxygen therapy	16,145 (53%)	14,562 (54%)	1,583 (51%)	0.001
Nasal cannula	281 (0.9%)	236 (0.9%)	45 (1.4%)	0.002
Admission to high care unit	1,257 (4.1%)	1,040 (3.8%)	217 (6.9%)	< 0.001
Admission to intensive care unit	2,753 (9.1%)	2,388 (8.8%)	365 (12%)	< 0.001
Non-open chest compression	104 (0.3%)	97 (0.4%)	7 (0.2%)	0.3
Continuous hemodiafiltration	142 (0.5%)	132 (0.5%)	10 (0.3%)	0.2
Ventricular assist device implantation	76 (0.3%)	69 (0.3%)	7 (0.2%)	0.9
Countershock	508 (1.7%)	456 (1.7%)	52 (1.7%)	> 0.9
Hospital bed volume (tertile), n (%)				0.094
1	10,103 (33%)	9,023 (33%)	1,080 (35%)	
2	10,037 (33%)	8,983 (33%)	1,054 (34%)	
3	10,156 (34%)	9,160 (34%)	996 (32%)	

CR, cardiac rehabilitation; NYHA, New York Heart Association; SD, standard deviation; ADL, Activities of Daily Living.

**Table 2:** Cardiac rehabilitation performed during the study period.

	Overall *n* = 30,296	Delayed or no CR *n* = 27,166	Early CR *n* = 3,130
CR prescription, n (%)	10,470 (35%)	7,340 (27%)	3,130 (100%)
CR sessions (total), mean (SD)	6 (17)	4 (16)	18 (25)
Average CR sessions per day, mean (SD)	0.43 (0.77)	0.32 (0.68)	1.36 (0.84)
CR days, mean (SD)	4 (10)	3 (10)	12 (13)

CR, cardiac rehabilitation; SD, standard deviation.

**Table 3:** Patient characteristics after propensity score matching and multiple imputation (dataset no. 1).

	Overall *n* = 6,258	Delayed or no CR *n* = 3,129	Early CR *n* = 3,129	*P*-value
Sex, n (%)				
Men	4,278 (68.4%)	2,140 (68.4%)	2,138 (68.3%)	0.978
Women	1,980 (31.6%)	989 (31.6%)	991 (31.7%)	
Age, year n (%)				0.995
18–64	2,133 (34.1%)	1,054 (33.7%)	1,079 (34.5%)	
65–69	842 (13.5%)	424 (13.6%)	418 (13.4%)	
70–74	833 (13.3%)	421 (13.5%)	412 (13.2%)	
75–79	970 (15.5%)	488 (15.6%)	482 (15.4%)	
80–84	786 (12.6%)	390 (12.5%)	396 (12.7%)	
85–89	549 (8.8%)	279 (8.9%)	270 (8.6%)	
≥ 90	145 (2.3%)	73 (2.3%)	72 (2.3%)	
NYHA classification, n (%)				0.963
Ⅱ	1,693 (27.1%)	844 (27.0%)	849 (27.1%)	
Ⅲ	2,615 (41.8%)	1,305 (41.7%)	1,310 (41.9%)	
Ⅳ	1,950 (31.2%)	980 (31.3%)	970 (31.0%)	
Impaired consciousness, n (%)	5,754 (91.9%)	2,870 (91.7%)	2,884 (92.2%)	0.546
Body mass index, kg/m^2^, n (%)				0.922
< 18.5	909 (14.5%)	449 (14.3%)	460 (14.7%)	
18.5–24.9	3,586 (57.3%)	1,798 (57.5%)	1,788 (57.1%)	
≥ 25	1,763 (28.2%)	882 (28.2%)	881 (28.2%)	
Charlson comorbidity index, n (%)				0.841
2	4,593 (73.4%)	2,302 (73.6%)	2,291 (73.2%)	
3	1,169 (18.7%)	576 (18.4%)	593 (19.0%)	
≥ 4	496 (7.9%)	251 (8.0%)	245 (7.8%)	
Smoking index, mean (SD)	323 (917)	316 (542)	329 (1178)	0.591
ADL score, mean (SD)	14.43 (7.44)	14.45 (7.48%)	14.41 (7.40)	0.854
Ambulation, n (%)	1,613 (25.8%)	789 (25.2%)	824 (26.3%)	0.326
Unexpected admission, n (%)	4,492 (71.8%)	2,247 (71.8%)	2,245 (71.7%)	0.978
Pharmaceuticals within 3 days of admission, n (%)				
Carperitide	2,096 (33.5%)	1,043 (33.3%)	1,053 (33.7%)	0.81
Nitrate	882 (14.1%)	435 (13.9%)	447 (14.3%)	0.689
Nicorandil	51 (0.8%)	27 (0.9%)	24 (0.8%)	0.779
Ca-channel blocker	214 (3.4%)	111 (3.5%)	103 (3.3%)	0.626
Dopamine	329 (5.3%)	156 (5.0%)	173 (5.5%)	0.365
Dobutamine	1,425 (22.8%)	704 (22.5%)	721 (23.0%)	0.63
Milrinone	203 (3.2%)	100 (3.2%)	103 (3.3%)	0.887
Noradrenaline	89 (1.4%)	45 (1.4%)	44 (1.4%)	1
Adrenaline	30 (0.5%)	19 (0.6%)	11 (0.4%)	0.2
Pimobendane	931 (14.9%)	482 (15.4%)	449 (14.3%)	0.256
Furosemide	3,368 (53.8%)	1,687 (53.9%)	1,681 (53.7%)	0.899
Tolvaptan	1,386 (22.1%)	678 (21.7%)	708 (22.6%)	0.377
Procedures within 3 days of admission, n (%)				
Intra-aortic balloon pumping	17 (0.3%)	10 (0.3%)	7 (0.2%)	0.627
Endotracheal mechanical ventilation	1,054 (16.8%)	528 (16.9%)	526 (16.8%)	0.973
Oxygen therapy	3,200 (51.1%)	1,617 (51.7%)	1,583 (50.6%)	0.404
Nasal cannula	94 (1.5%)	50 (1.6%)	44 (1.4%)	0.603
Admission to high care unit	419 (6.7%)	203 (6.5%)	216 (6.9%)	0.544
Admission to intensive care unit	723 (11.6%)	358 (11.4%)	365 (11.7%)	0.812
Non-open chest compression	19 (0.3%)	12 (0.4%)	7 (0.2%)	0.358
Continuous hemodiafiltration	17 (0.3%)	7 (0.2%)	10 (0.3%)	0.627
Ventricular assist device implantation	11 (0.2%)	4 (0.1%)	7 (0.2%)	0.546
Countershock	109 (1.7%)	57 (1.8%)	52 (1.7%)	0.699
Hospital bed volume (tertile) n (%)				0.495
1	2,189 (35.0%)	1,110 (35.5%)	1,079 (34.5%)	
2	2,065 (33.0%)	1,011 (32.3%)	1,054 (33.7%)	
3	2,004 (32.0%)	1,008 (32.2%)	996 (31.8%)	

CR, cardiac rehabilitation; NYHA, New York Heart Association; SD, standard deviation; ADL, Activities of Daily Living.

**Table 4:** Outcomes on propensity score matching method with multiple imputation.

	Odds ratio [95% CI]	*P*-value
90-day mortality	0.70 [0.53–0.93]	0.01
	Mean difference [95% CI]	P-value
ADL score at discharge	0.43 [0.08–0.78]	0.02
Length of stay	−2.12 [−4.72–0.48]	0.11

CI, confidence interval; ADL, Activities of Daily Living.

**Table 5:** Descriptive statistics for various outcomes.

	Overall *n* = 30,296	Delayed or no CR *n* = 27,166	Early CR *n* = 3,130
90-day mortality, n (%)	1,714 (5.7%)	1,587 (5.8%)	127 (4.1%)
ADL score, mean (SD)	17.11 (6.17)	17.06 (6.22)	17.46 (5.65)
Missing, n	2,161	1,929	232
Length of stay, mean (SD)	28 (44)	28 (46)	25 (27)

CR, cardiac rehabilitation; ADL, Activities of Daily Living; SD, standard deviation.

The results for patients with NYHA classification Ⅳ and the changed definition of early CR were similar to those of this analysis. In contrast, stratified analyses of those with NYHA classifications Ⅱ and Ⅲ showed no significant differences with respect to any outcomes (Tables 6 and 7).

**Table 6:** Outcomes on propensity score matching method with multiple imputations for patients with DCM (NYHA classification Ⅱ, Ⅲ, Ⅳ).

		Odds ratio [95% CI]	P-value
NYHA classification Ⅳ	90-day mortality	0.62 [0.43–0.88]	0.01
NYHA classification Ⅲ	90-day mortality	0.72 [0.44–1.28]	0.19
NYHA classification Ⅱ	90-day mortality	1.20 [0.54–2.69]	0.66

		Mean difference [95% CI]	P-value
NYHA classification Ⅳ	ADL score at discharge	1.04 [0.35–1.74]	0.003
	Length of stay	−4.53 [−9.29–0.23]	0.06
NYHA classification Ⅲ	ADL score at discharge	0.33 [−0.17–0.82]	0.20
	Length of stay	−2.06 [−5.03–0.91]	0.17
NYHA classification Ⅱ	ADL score at discharge	−0.16 [−0.68–0.35]	0.53
	Length of stay	0.77 [−2.65–4.18]	0.66

NYHA, New York Heart Association; CI, confidence interval; ADL, Activities of Daily Living.

**Table 7:** Outcomes on propensity score matching method with multiple imputation for patients with DCM (early CR defined as “CR initiated within 48 h after admission”).

	Odds ratio [95% CI]	*P*-value
90-day mortality	0.70 [0.50–0.98]	0.04

	Mean difference [95% CI]	*P*-value
ADL score at discharge	0.47 [0.03–0.91]	0.04
Length of stay	−2.05 [−4.82–0.71]	0.15

DCM, dilated cardiomyopathy; CR, cardiac rehabilitation; CI, confidence interval; ADL, Activities of Daily Living.

## Discussion

We used a national inpatient database to investigate the association of early CR with the short-term prognosis of patients with DCM using propensity score matching. To our knowledge, this is the first study to examine the association between early CR and outcomes in adult patients with HF of all ages diagnosed with DCM, using a large-scale dataset. The results showed a lower 90-day mortality and higher ADL scores at discharge in the early CR group compared to the delayed or no CR group. These results suggest that early CR may improve the short-term prognosis of patients with DCM.

Using the DPC database, we analyzed a specific group of patients with symptomatic HF for whom the influence of CR is under-researched. For example, the recent REHAB-HF trial assessed early CR in older patients hospitalized for acute decompensated HF, with significantly enhanced physical function following early commencement and gradual transition to personalized CR^39^. However, it did not explore the variability in effects by disease type, such as DCM. Our study bridges this gap. Subgroup analysis by the NYHA classification revealed that early CR improves prognoses across NYHA classifications Ⅱ–Ⅳ. CR programs with appropriate risk management may improve physical and mental function by minimizing problems associated with excessive bed rest (e.g., disuse syndrome), common in patients hospitalized regardless of their symptoms. Since Schweickert WD et al. reported in 2009 that early mobilization for patients with mechanical ventilation improves patients’ prognosis, early mobilization has attracted attention as an intervention that can improve prognosis^40^. However, it has recently become clear that frailty and multiple comorbidities are prognostic factors for older patients with HF, and early mobilization for patients with acute HF is increasingly attracting attention as an intervention that can improve prognosis by preventing disuse syndrome, frailty, and multiple comorbidities^14,39,41,42^. Okamura M et al. conducted a systematic review of early mobilization for patients with acute HF, found no applicable RCTs, determined that two observational studies were eligible, and as a result of integration found that the early mobilization group had the potential to significantly reduce rehospitalization rates compared to the control group (283 participants in total; OR: 0.25, 95% CI: 0.14 − 0.42; I^2^ = 0%; low-certainty evidence)^42^. Although clarifying the mechanisms underlying the improved 90-day mortality and ADL scores at discharge remains challenging, a previous small study reported that CR enhanced coronary endothelial function in patients with stable non-ischemic DCM^43^. Enhanced myocardial perfusion from CR may improve patients’ circulatory function and physical performance. Furthermore, prospective studies on exercise interventions in patients with DCM have consistently shown improvements in functional capacity and HRQoL^23,44–46^. Although our study focused on patients with DCM and acute HF, exercise interventions, including early mobilization under appropriate supervision, may have contributed to the prevention of disuse syndrome and improvement of functional capacity^47^, potentially leading to a modest enhancement in ADL score at discharge. The ADL score used in this study is the Barthel Index (BI) ADL score, with a total score of 20 points, used by Collin et al.^48^. Reports of MCID for this ADL score are very limited; however, Bouwstra H et al.’s study on older patients admitted to a nursing home in the Netherlands reported that the clinical-based minimal important change (MIC) of BI for return home or not was 3.1 (95% CI: 2.0 − 4.2), and the patient-based MIC was 3.6 (95% CI: 2.8 − 4.3)^49^. However, Katano S et al. used Cox regression analysis to examine factors affecting patient mortality in older patients with HF who underwent CR during hospitalization and reported that BI at discharge (on a 100-point scale) was an independent factor associated with mortality^50^. Motoki H et al. also reported the impact of BI at discharge (on a 100-point scale) on post-discharge mortality after CR during hospitalization for patients with acute decompensated HF and showed that better outcomes were observed in patients who had a BI improvement of 15 points or more before and after hospitalization^51^. Although there was no directly usable evidence on MCID for the ADL scores used in this study due to differences in the patients and scores used, the degree of improvement in ADL scores with early CR in this study was relatively small; it was not large enough to contribute significantly to long-term outcomes, such as post-discharge mortality. However, approximately 60% of the present study’s participants were older adults, acutely hospitalized patients with DCM with HF symptoms. Therefore, because of their age and pathological characteristics, the participants in this study were unlikely to achieve dramatic improvement in ADL levels following hospitalization. We also cannot rule out the possibility that some of the patients in this study had already experienced a decline in their ADL scores prior to hospitalization.

This study has some limitations. First, the DPC database does not include echocardiographic or physiological tests, restricting the adjustment for confounding factors. Second, intervention was defined based on whether CR was initiated within 3 days of admission; variations in intervention content may have influenced the estimated associations. Third, we focused on short-term prognostic outcomes, such as ADL at discharge and 90-day mortality. Other outcomes related to physical and mental functioning (e.g., HRQoL at discharge, muscle strength, or dyspnea on exertion) and negative outcomes (e.g., adverse events during hospital stay) were not included in our analysis. Nonetheless, this study is the first to investigate the association of early CR with the outcomes in patients with symptomatic DCM.

In summary, our study used the DPC database and the 2015 Hospital Bed Function Report and found that early CR for patients with DCM was associated with lower 90-day mortality and slightly higher ADL score at discharge, especially for those with moderate or severe symptoms. Further research is needed to elucidate the effectiveness of early CR and its physiological mechanisms in patients with DCM and to develop a more personalized and effective CR program.

## Data Availability

The datasets analyzed in this study are not publicly available because of contracts with the hospitals that provide data to the database. However, they are partially available from the corresponding author upon reasonable request.
